# Effective Diffusion in Fibrous Porous Media: A Comparison Study between Lattice Boltzmann and Pore Network Modeling Methods

**DOI:** 10.3390/ma14040756

**Published:** 2021-02-05

**Authors:** Xiang Huang, Wei Zhou, Daxiang Deng

**Affiliations:** 1 Fujian Key Laboratory of Special Energy Manufacturing, Xiamen Key Laboratory of Digital Vision Measurement, Huaqiao University, Xiamen 361021, China; 2 Department of Mechanical & Electrical Engineering, Xiamen University, Xiamen 361005, China; weizhou@xmu.edu.cn; 3 Harbin Institute of Technology, School of Mechanical Engineering and Automation, Shenzhen 518055, China; dengdaxiang@hit.edu.cn

**Keywords:** porous media, pore network modeling, lattice Boltzmann method, diffusion, tortuosity

## Abstract

The understanding of the correlation between a pore-scale structure and its coupled diffusion transport property is crucial in the virtual design and performance optimization of porous fibrous material for various energy applications. Two most common and widely employed pore-scale modeling techniques are the lattice Boltzmann method (LBM) and the pore network modeling (PNM). However, little attention has been paid to the direct comparison between these two methods. To this end, stochastic porous fibrous structures are reconstructed reflecting the structural properties of the fibrous porous material on a statistical level with structural properties obtained from X-ray computed microtomography. Diffusion simulation through the porous phase was subsequently conducted using LBM of D3Q7 lattice and topological equivalent PNM derived from the watershed method, respectively. It is detected that the effective diffusion coefficients between these two methods are in good agreement when the throat radius in the pore network is estimated using the cross-section area equivalent radius. Like most literature, the diffusivity in the in-plane (IP) direction is larger than in the through-plane (TP) direction due to the laid fiber arrangement, but the values are very close. Besides, tortuosity was evaluated from both geometry and transport measurements. Tortuosity values deduced from both methods are in line with the anisotropy of the diffusion coefficients.

## 1. Introduction

Fibrous porous materials are widely applied for various industrial applications, such as the gas diffuse layer [1,2], catalyst support layer [3,4], filter [5,6], and wipes [7,8]. The transport properties are strongly influenced by the porous architecture, such as porosity, pore size distribution, connectivity, tortuosity, etc. Therefore, a complete understanding of the correlation between the transport property and porous morphology would greatly help improve the accurate evaluation and optimization of a material structure for various applications.

To determine the transport property in the porous material, with a particular focus on the effective diffusion coefficient, several in-situ [9] and ex-situ [10] techniques have been developed. For a systematic review, the readers are suggested to refer to Reference [11]. Generally, these experimental reports provided fundamental insight into the mass transport property. However, due to the small length scale of the porous structure, the experimental measurements of effective properties are not always easy to conduct. Moreover, most of the experimental measurements focus on the commercial gas diffuse layer, which was generally fabricated with carbon fibers attached with polytetrafluoroethylene (PTFE) and binders to generate the hydrophobic and unique porous architecture. Therefore, the measured property cannot truly reflect the property of general fibrous porous structures without additional content.

Alternatively, numerical simulation is a widely used technique that provides the possibility of understanding between various fibrous porous structures and a coupled transport property. Combining X-ray tomographic images with the Lattice Boltzmann Method (LBM) implemented using the parallel open-source LBM solver Palabos (https://palabos.unige.ch/), Salaberri [12,13,14] investigated the effective diffusion coefficients of dry and partially saturated carbon-fiber gas diffusion layers (GDL). Calculations on dry GDLs were in close agreement with previous experimental data. The heterogeneity of the microstructure was evaluated through simulations of different subdomains of computerized tomography (CT) images, and the existence of a representative elementary volume (REV) was assessed in terms of dry, effective transport properties. A so-called M-factor [15] was introduced to describe the impact of a GDL microstructure on the effective transport property. The functional properties of the virtual fibrous structure were investigated using commercial software Geodict (http://www.geodict.com). The Micro-CT scanning technique can be very expensive and time-consuming. Therefore, the digitally stochastic model with the 3D quantification of the microstructure from 3D images is often employed as an alternative. Yiotis [16] proposed a stochastic reconstruction methodology of GDL with structural properties obtained from X-ray computed microtomography, the effective transport properties such as permeability, diffusivity, thermal/electrical conductivity, and void tortuosity were then determined. Zamel [17] studied the effective diffusion of GDL with the 3D stochastic model generated using Geodict. The generated mesh was later imported into the commercial computer fluid dynamics (CFD) solver Fluent. Stochastic 3D fibrous models were also constructed and numerically studied in similar works [17,18] using different software AVIZO (FEI, Waltham, MA, USA) [18] and OpenFOAM (ESI, Paris, France) [19]).

Pore network modeling (PNM) idealizes the complex pore space as a concise representation of the network comprised of pores connected by narrow throats and is now drawing increasing attention of the research community due to its scale advantage and efficiency. Generally, the structure of the pore network can be constructed regularly as lattice [20,21,22,23] or topologically equivalent using image processing [24,25], where the latter type is the focus in our work. Chevalier et al. [26] customized the watershed derived PNM and explored the effective in-plane (IP) diffusivities using the open-source package OpenPNM [27]. The validation of the extracted pore network was verified using the experimental data. Similarly, when Banerjee et al. [28] directly applied the pore network extraction module within the OpenPNM framework, the calculated transport property was in agreement with the experimental one. Notably, the capacity of PNM is expanding to not only evaluate the transport property including invasion percolation and diffusion but also reaction [29], condensation, and evaporation [30] were implemented.

Many correlations have been proposed to estimate the transport properties affected by porosity and saturation. Among them, the Bruggeman approximation [17], which was initially proposed for porous media composed of spherical particles but expressed with the most concise formulation, was widely applied for curve fitting. However, the fitting parameter of the Bruggeman equation varies in the existing literature [14,19,26], and results in the dispersion of the degree of anisotropy between the in-plane (IP) and through-plane (TP) diffusivity. Moreover, a parametric study of the impact of the throat radius on the percolation invasion was performed by the author of OpenPNM [31]. However, its impact on the effective diffusion was not seen.

In this work, a promising porous fibrous material, known as a porous metal fiber sintered sheet (PMFSS), which has been successfully applied in fuel cells as a catalyst support layer [32] and a wipe in a heat pipe [33], was investigated for its effective diffusivity. The effective diffusion in the IP and TP directions using both LBM and PNM methods are performed and compared, along with the tortuosity deduced from the transport and morphological measurements. This work aims to explore the impact of a fibrous, porous microstructure on the effective diffusivity and, meanwhile, verify the proper parameter setting of PNM, which can finally provide guidelines for designing optimized fibrous porous structures.

## 2. Materials and Methods

### 2.1. Materials

**Figure 1:**
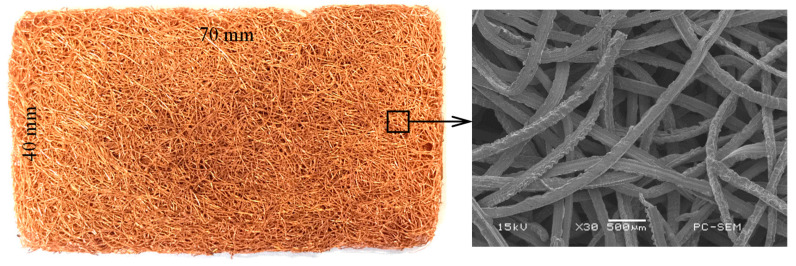
Photograph of 80% porosity porous metal fiber sintered sheet (PMFSS) sizing of 70 × 40 × 2 mm^3^. The SEM image on the right shows the magnified microstructure.

PMFSS is composed of copper fibers cut using a specially designed homemade multi-tooth turning tool. Continued fibers were later cut into the fixed-length, loaded into a mold, and pressed until desired porosity was reached. PMFSS was eventually fabricated after a solid-phase sintering method [34]. Figure 1 shows the photo and Scanning Electron Microscope (SEM) image of an 80% porosity PMFSS sample with a size of 70 × 40 × 2 mm^3^. It can be seen that the PMFSS exhibited a 3D fiber network architecture, which is composed of curved fibers with a mean radius of about 50 μm almost aligning horizontally in the material plane.

### 2.2. Microstructure Reconstruction

**Figure 2:**
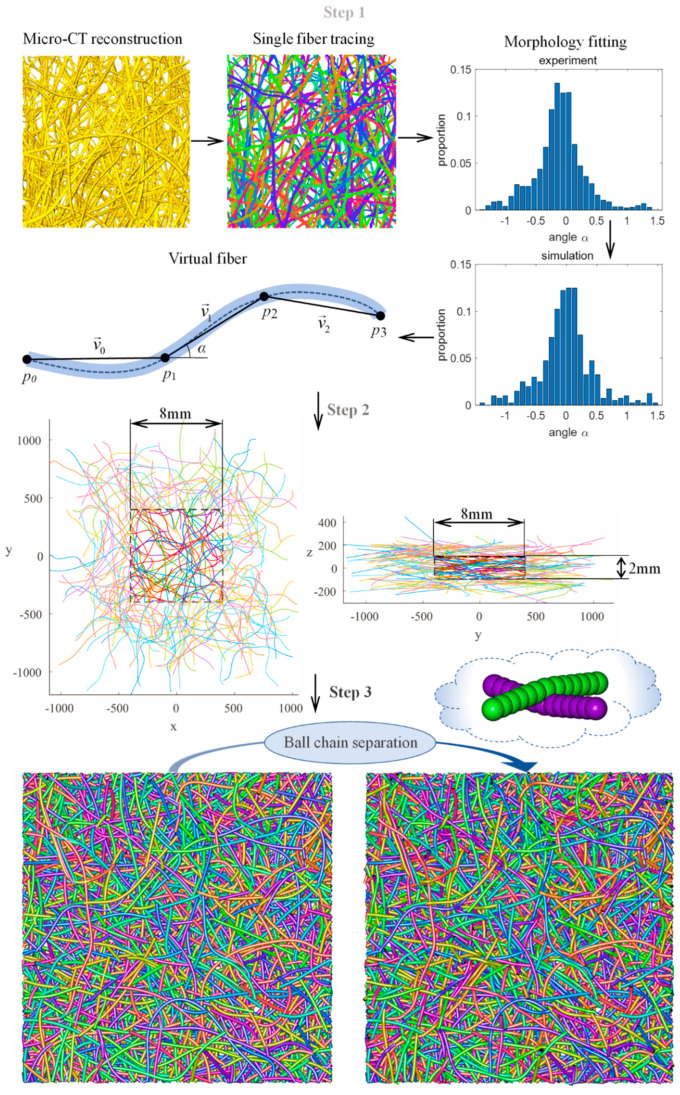
Generation of virtual PMFSS divided into three stages. Step 1: single fiber simulation, step 2: fiber accumulation, and step 3: fiber separation.

Figure 2 shows the modeling process of virtual PMFSS, which can be briefly divided into three steps: (1) single fiber generation based on statistical distribution information obtained from micro-CT images (HARRIER HP23.4JX, Metris UK Systems LTD), (2) fiber mat generation as accumulation of single fibers, and (3) non-overlapping fiber system conversion using ball chain simulation. In this work, curved spline fibers of about an 8-mm physical length are randomly orientated in the in-plane direction and obeying through-plane orientation distribution of measured experimental data. The center point of each fiber is randomly distributed in a box of a physical size of 16 × 16 × 2 mm^3^ (x × y × z), and the core section of a box size of 8 × 8 × 2 mm^3^ is then cut out as the simulation domain (Figure 2, step 2), which is later treated using ball chain simulation to separate overlapping fibers (step3). Finally, the 3D image of virtual PMFSS is generated by dilating the centerline of separate fibers with a sphere of fiber radius using Matlab (MathWorks, Natick, MA, USA). By varying the number of fibers, desired porosity can be obtained, which can be measured as the ratio of voxel numbers in the binary image accordingly. Following these steps, four models with porosities of 61%, 68%, 79%, and 90% were generated with initially 6400, 4650, 3047, and 1550 fibers laid in the 16 × 16 × 2 mm^3^ modeling domain. For technical details such as fiber tracing, virtual fiber mat generation, and ball chain separation, readers are suggested to view our former reports [35,36].

### 2.3. Pore Network Modeling Simulation

**Figure 3:**
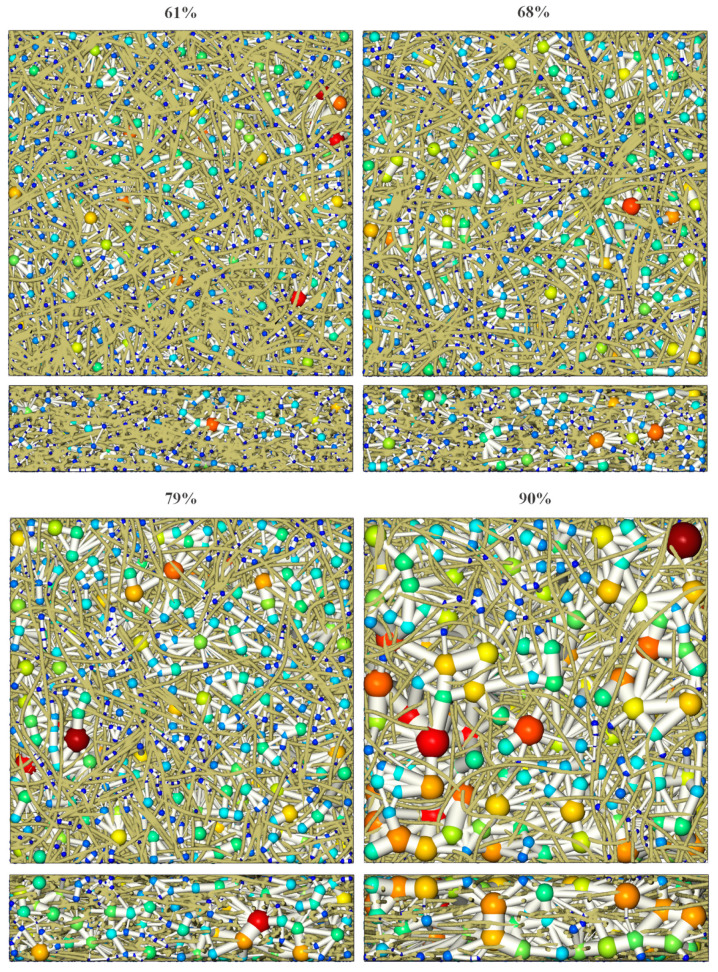
Pore network modeling (PNM) of 61%, 68%, 79%, and 90% porosity porous metal fiber sintered sheet (PMFSS).

A watershed-derived pore network extraction based on open-source software “imorph” was applied [37]. Figure 3 shows the extracted pore network inside the reconstructed 3D fiber system. The spheres are shown using the jet colormap to tell the varied pore sizes from small (dark blue) to big (dark red) sizes. The more detailed distribution data is demonstrated in Section 3.1. It can be seen that the void space of virtual PMFSS was successfully divided into individual spherical pore cells connected by cylindrical throats. Note that the pore cell is rendered using a 3D ball of center point with the local maximal distance value. Similar treatment is applied to the throat, where two neighboring pore cells are connected with a cylinder of centerline connecting the center points of the two-neighboring ball-representing pore cells. The cylinder is rendered with a radius equal to the maximal distance value at the interface between the two neighboring pore cells. On closer observation, especially at 90% porosity, it can be seen that some throats are penetrated by fibers. It happens since the flow paths are tortuously expanded to avoid fibers rather than straightness, as assumed in PNM.

**Figure 4:**
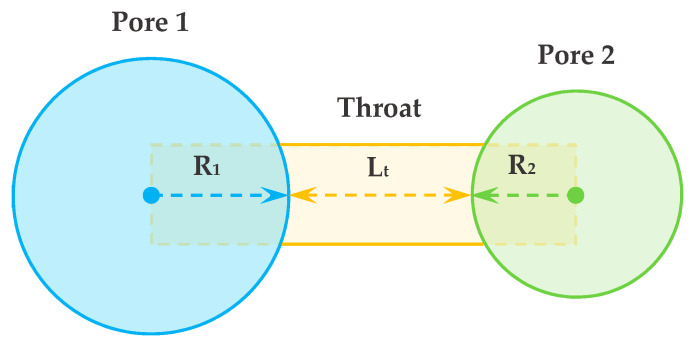
Illustration of pore and throat configuration in the pore network used for diffusion simulation.

Once the pore network is generated, the diffusive transport within the pore space is determined. The molar flow of gas between two pores connected by a throat (Figure 4) can be written as:

(1) $$j_{1,2}=g_{1,2}\left(x_{p1}-x_{p2}\right)$$

where *g*_1,2_ is the diffusion conductivity and *x_pi_* is the mole fraction (concentration) of pore *i*. Taking account of the impact of structures of the throat and pore, the diffusive conductance was calculated as follows:

(2) $$g_{1,2}={\left(\frac{1}{g_{t}}+\frac{1}{g_{p1}}+\frac{1}{g_{p2}}\right)}^{-1}$$

where *g_pi_* is the diffusive conductance of pore *i*, and *g_t_* is the conductance of throat *t*. The diffusive conductivity of pore is defined as:

(3) $$g_{pi}=CD_{b}\frac{A_{pi}}{R_{pi}}$$

where *C* is the molecular density of the gas, *D_b_* is the bulk diffusivity (open-space diffusivity without obstacle), *A_pi_* is the cross-section area of the pore *i*, and *R_pi_* is the radius of pore *i*. Similarly, the diffusive conductivity of the throat is:

(4) $$g_{t}=CD_{b}\frac{A_{t}}{L_{t}}$$

where *A_t_* is the cross section area of the throat, and *L_t_* is the length of the throat, which equals the distance between sphere centers minus their radius.

The characteristic length of the fibrous system is of a micrometer magnitude, which is much bigger than the mean free path of the gas molecules. Therefore, Knudsen diffusion is insignificant. By solving mass conservation on each pore by applying Equations (1)–(4), a linear system is constructed, where the mole fraction of gas in each pore can be resolved. The normalized effective diffusivity of the modeling system is computed using Fick’s law as the ratio between the diffusive flux in the direction of interest and the bulk flux as follows:

(5) $$\frac{D_{eff}}{D_{b}}=\frac{jL}{D_{b}\left(x_{in}-x_{out}\right)CA}$$

where *L* is the length of the simulation domain, *A* is the cross-section area, and *j* is the total molar flow through the domain, which could be obtained at the inlet or outlet. *x_in_* and *x_out_* are the gas concentration in the Dirichlet boundary conditions. The lateral sides of the simulation domain are treated as wall conditions with no molar flow rate. The values of parameters in Equations (1)–(5) can be assigned as suggested in Reference [26], where properties of oxygen at room temperature were used. Note that the values of these parameters (*C*, *D_b_*, *x_in_*, and *x_out_*) will not change the result of the normalized effective diffusivity calculation.

### 2.4. Lattice Boltzmann Simulation

A D3Q7 lattice with the standard single relaxation time Bhatnagar-Gross-Krook (BGK) collision operator was implemented to explore the gas diffusive transport within virtual PMFSS. The governing equation of the model is expressed as [14]:

(6) $$f_{\alpha }\left(x_{i}+c_{\alpha }\Delta t,t+\Delta t\right)-f_{\alpha }\left(x_{i},t\right)=-\frac{f_{\alpha }\left(x_{i},t\right)-f_{\alpha }^{eq}\left(x_{i},t\right)}{\tau },\;\alpha =0,\ldots ,6$$

where *fα* (*x_i_*, *t*) is the particle distribution at location *x_i_* and time *t*, *Δt* (=1) is the time step, *τ* (=1) is the dimensionless relaxation time, and *c*_α_ and *f*_α_*^eq^* are the velocity and equilibrium population.

(7) $$c_{\alpha }=\{\begin{array}{cc} \left(0,0,0\right)\; & \alpha =0\\ \left(\pm 1,0,0\right),\left(0,\pm 1,0\right),\left(0,0,\pm 1\right) & \alpha \neq 0 \end{array},\;f_{\alpha }^{eq}=\{\begin{array}{cc} \left(1-3c_{s}^{2}\right)C & \alpha =0\\ \left(c_{s}^{2}/2\right)C & \alpha \neq 0 \end{array}$$

where *C* is the macroscopic concentration and *c_s_* = 1/2 is the lattice speed of sound. The macroscopic concentration *C*, bulk diffusivity (*D_b_*), and diffusion molar flux (*j*) are obtained as:

(8) $$C=\overset{6}{\underset{\alpha =0}{\sum }}f_{\alpha },\;D_{b}=c_{s}^{2}\left(\tau -\frac{1}{2}\right),\;j=\left(1-1/2\tau \right)\overset{6}{\underset{\alpha =0}{\sum }}f_{\alpha }c_{\alpha }$$

A concentration difference between 1 and 0 is arbitrarily imposed at the inlet and outlet surfaces applying a Zou and He boundary condition [38] (*ΔC* = 1), which can be easily deduced by a mass conservation criterion. Note that the values of the inlet and outlet boundary will not change the result of the normalized effective diffusivity. The lateral sides and the interface between the pore and fibers are treated as a wall boundary using a half-way bounce back scheme, just like the set-up of PNM. Finally, according to Fick’s law, the normalized effective diffusivity is obtained as:

(9) $$\frac{D_{eff}}{D_{b}}=\frac{(\int_{A}j_{i}dA)/A}{D_{b}\Delta C/L}$$

where *j_i_* is the diffusion flux component in the simulation direction (IP or TP). It is notable that, since the lateral surfaces are treated as walls with no molar flow rate, the value of parameter *j_i_* is almost equal to the total flux *j*, in Equation (5), through the simulation domain. The LBM simulation was implemented using c++ code based on LBM project named ‘lb3d’ (https://code.google.com/archive/p/lb3d-prime-dev/).

## 3. Results and Discussion

### 3.1. Pore Network Analysis

**Figure 5:**
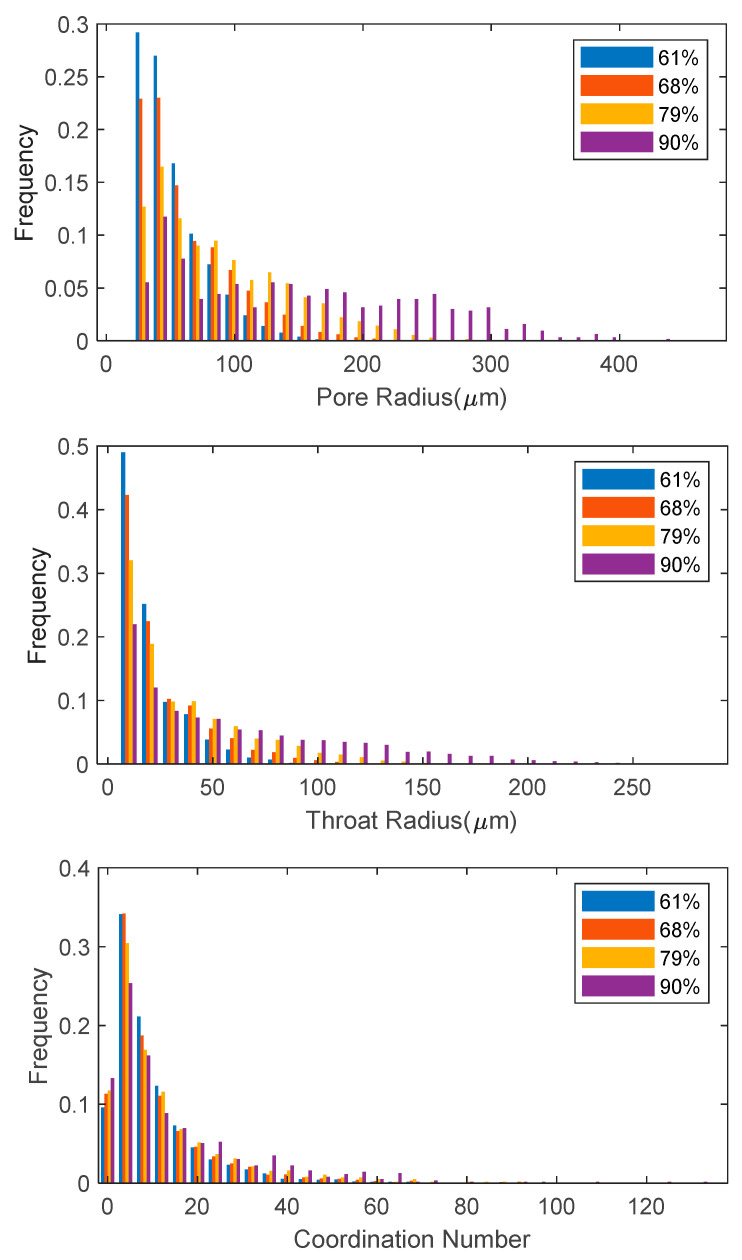
Distributions of pore radius, throat radius, and coordination number in 61%, 68%, 79%, and 90% porosity PMFSS.

The impact of porosity on the distributions of the pore and throat radius distribution as well as the coordination number, which equals the number of throats connected to a pore, are presented in Figure 5. The mean values are listed in Table 1. As the porosity increases from 61% to 90%, the distributions of the pore and throat radius both shift right and the mean pore radius increases from 60.2 μm to 157.1 μm. In contrast, the mean throat radius is smaller, ranging from 23.3 μm to 65.4 μm. The distribution of the coordination number was found to be relatively constant regardless of the change of porosity and the mean coordination number was found to slightly increase as the porosity increases. This effect was expected since larger pore cells will meet more neighboring ones for higher porosity.

### 3.2. Comparison of Effective Diffusivity Using LBM and PNM

The image resolution used for PNM is 400 × 400 × 100 (x × y × z), considering the physical size (8 × 8 × 2 mm) and fiber diameter (0.1 mm), that is, there are about five pixels in the fiber’s width. The pore network extraction takes less than 1 h on the laptop (CPU i7-9750H, RAM 16 G), depending on the complexity of the domain (model of lower porosity takes more time since there are more pores to consider, as shown in Figure 3). Diffusion calculations are completed within 5 s. The LBM simulation takes a long time. Thus, to save the computational resource, the 3D image simulation domain is resampled by merging 8 pixels into 1, yielding an image with a resolution of 200 × 200 × 50. The simulation under this resolution takes several days (2 to 5) to reach the steady state (the relative error of molar fluxes between the inlet and outlet surfaces is within 0.1%), depending on the porosity (model of lower porosity takes a longer time since there are more lattice nodes to calculate) and flow direction (the in-plane simulation takes more time due to the longer flow pathway).

**Figure 6:**
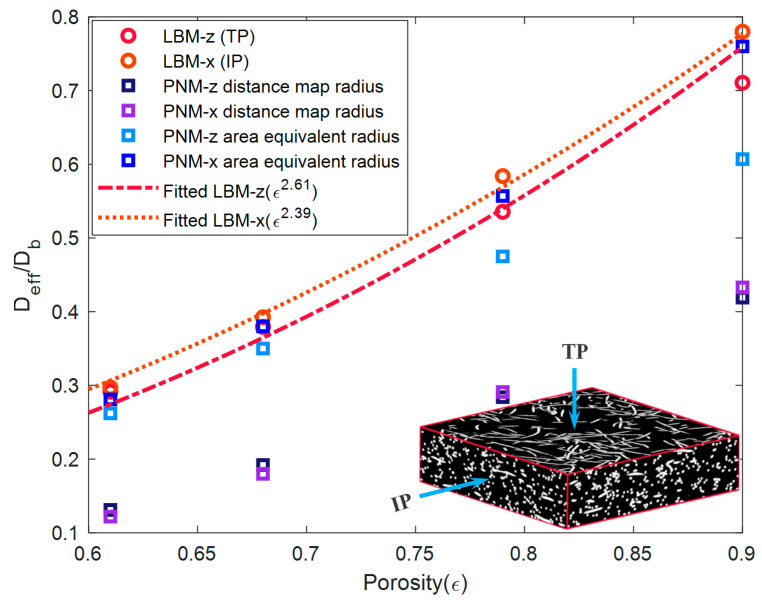
Dimensionless diffusivity of PMFSS in TP and IP directions obtained using PNM and LBM, and the fitted curve as a function of porosity.

**Figure 7:**
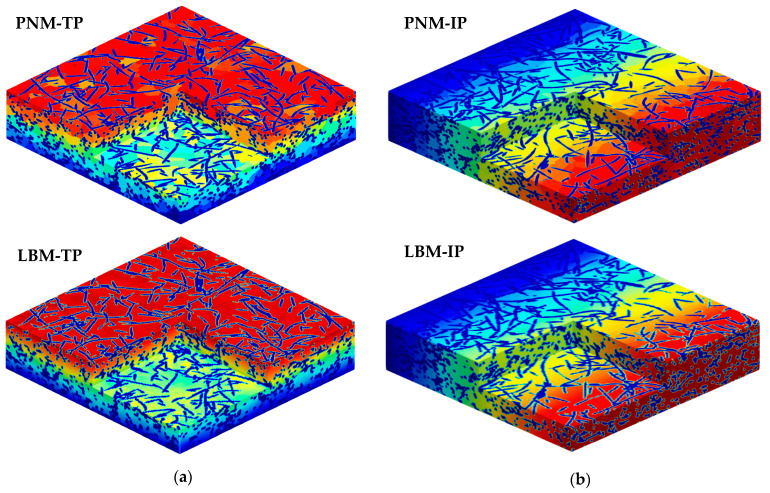
Concentration distribution of the 68% porosity PMFSS in left (**a**) TP and right (**b**) IP directions using PNM (top) and LBM (bottom) methods.

Relative to PNM, LBM takes a full account of the impact of the microstructure on the transport properties. Even though it is more time consuming, it can provide reliable results, and, thus, taken as the benchmark against which a parametric study of PNM is judged. Figure 6 shows the results of both the IP (x-direction) and TP (z-direction) directions. The direction of the x-axis is chosen without loss of generality since the fibers align isotropically in the material plane. In the extracted pore network, the throat is generated during watershed segmentation as a boundary between adjacent pores located at the chosen spots with the local maximal distance value. Therefore, the throats usually play as the narrow parts in the pore network architecture and result in the bottleneck during various mass transportations (Equation (2)). The cross-sectional shape of the throat is more complex than the inscribed circle with a radius of a local maximal distance value to characterize the throat size using the radius of the inscribed circle, which will lead to a conservative result. This problem is remedied by introducing the area-equivalent radius, i.e., a circle of area equivalent to the cross section’s radius, during the calibration of capillary pressure curves between the PNM result and the experiment measurement [31]. The mean value of an area-equivalent radius is about three times as much as the mean value of the distance map radius. In this work, the area-equivalent radius is also verified to be efficient for a diffusion calculation. A better agreement is achieved between PNM and LBM when the area-equivalent radius is used in Equation (4) for the throat section, and the distance map radius is assigned in Equation (3) for the pore section. It can be seen that the effective diffusivity is slightly higher in the IP direction due to the preferential alignment of fibers in the material plane. Taking a closer observation, it can be detected that the diffusivity values agree well in the IP direction using both methods. On the other hand, the diffusivity values in the TP directions show good agreement at lower porosities below 70%. However, the deviation turns higher at higher porosities and reaches about 15% for 90% porosity. In this work, we take this range of deviation as acceptable since most values still fit well using the simple area-equivalent radius strategy. The computed concentration fields of 68% porosity PMFSS using both methods are compared and shown in Figure 7. In each direction, the concentration distributions agree well for both methods. The continuous concentration distribution is demonstrated for LBM. In contrast, discrete distribution is shown for PNM since each pore cell is assigned with a certain concentration value.

### 3.3. Tortuosity

**Figure 8:**
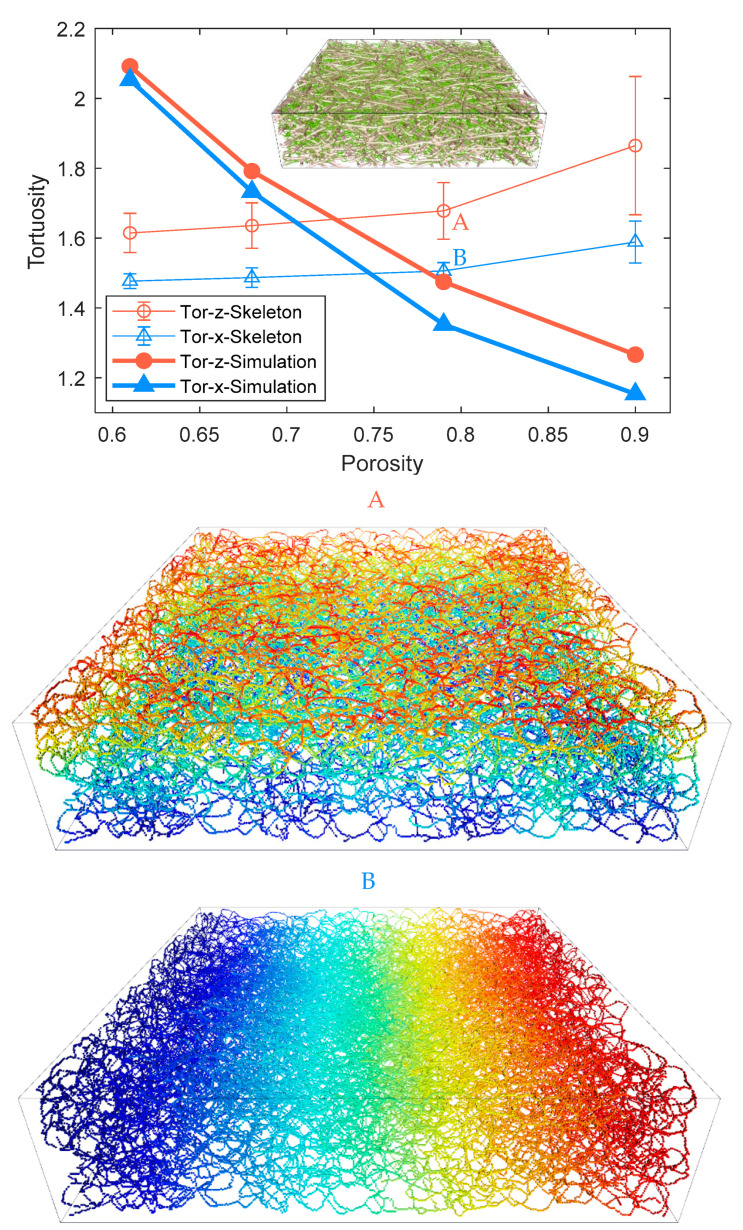
Comparation of tortuosity of 61%, 68%, 79%, and 90% porosity PMFSS, using both using Bruggeman approximation and a geometrical measurement. The colorful skeleton paths of pore phase of 79% porosity PMFSS shown in the middle (**A**) and bottom (**B**) indicate the continuous increase of pathway length from the chosen inlet surface to the outlet one.

The correlation between porosity and effective diffusivity was further fitted by the power law of the Bruggeman approximation.

(10) $$\frac{D_{eff}}{D_{b}}=\varepsilon^{m}$$

where *m* is the Bruggeman exponent [17]. Note that the default value of *m* is 1.5, which is used to predict the diffusivity of porous material composed of uniformly distributed spheres. The fitted *m* value equals 2.39 in the IP direction and 2.61 in the TP direction in our work, respectively, which is in a range of available literature [19,39] (between 2 and 3). The fitted value in Reference [13] is larger (*m* = 3.8 for the TP direction). The bonders are added in the fibrous network in Reference [13], which might increase the tortuosity of the flow domain. The Bruggeman approximation is also widely applied in another form below.

(11) $$\frac{D_{eff}}{D_{b}}=\frac{\varepsilon }{\tau }$$

where *τ* is the tortuosity of the flow domain, which is commonly defined as the ratio of the length of flow path between the inlet and the outlet boundaries to the straight-line distance between these two boundaries. The tortuosity could also be geometrically interpreted by the extracted skeleton [35]. For each skeleton voxel, the shortest path along the extracted skeleton connected to the chosen inlet can be determined using the Dijkstra algorithm, yielding a distance field. The geometrical tortuosity is then measured at each voxel of the outlet surface shown as the statistical average with the deviation in Figure 8, along with transporting tortuosity, according to Equation (11). The colorful skeleton paths of the pore phase of 79% porosity PMFSS in the middle (A) and bottom (B) of Figure 8 indicate the continuous increase of pathway length from the chosen inlet surface to the outlet one. The tortuosity values range from 1.1 to 2.2 as porosity increases from 61% to 90% for both methods. The TP tortuosity is consistently higher than the IP direction for each porosity. It is attributed to the fact that the flow pathway is parallel to the material plane in the IP direction while being vertical in the TP direction. As porosity decreases, the tortuosity increases, which leads to the non-linear dependence of diffusion on the porosity, as reflected by Figure 6 and indicated by the transporting tortuosity shown in Figure 8. However, the geometrical tortuosity in Figure 8 shows an irrational trend of increase as porosity increases. Note that the transporting tortuosity is a notion suggested to reflect the comprehensive mechanism impacted by the microstructural feature, involving not only the route of the flow path but also the pore shape, the connectivity, and bottleneck effect. The number of skeleton branches is lower when porosity is higher, which reduces the chance of meeting a shorter path.

## 4. Conclusions

In this work, the effective diffusion of PMFSS was explored using PNM and LBM methods. Both methods have their merits and limits. The high efficiency of diffusion calculation made PNM a promising tool for the large-scale simulation. Yet, a parameter calibration is required for compensating the deviation caused by geometrical simplification. On the other hand, the LBM is physically reliable but time consuming. A group of PMFSS with different porosity was generated to explore the impact of a structural property on effective diffusion. The structural properties, involving a pore and throat radius, with coordinate numbers were analyzed. The results showed that the increase of porosity will lead to the increase of a mean pore and throat radius and coordination numbers. Most importantly, it was detected that the effective diffusion in IP and TP directions, using both methods, agrees well when the throat radius was characterized using the area-equivalent radius in PNM. The concentration distribution in IP and TP directions, yielding from both methods, were shown and compared. Their good agreement further establishes the reliability of PNM for large-scale transport property exploration. The effective diffusion in the IP direction is slightly higher than the TP direction, and it is in line with the structural anisotropy of the laid arrangement of fiber orientations. Moreover, the tortuosity of PMFSS pore space was studied via diffusion transport using Bruggeman approximation and geometrical measurement based on a skeleton extraction. Both methods show agreement in the anisotropy property of IP and TP directions.

## Figures and Tables

**Table 1:** Effect of porosity on the mean values of pore and throat radius and coordination number.

Porosity	Mean Pore Radius (μm)	Mean Throat Radius (μm)	Mean Coordination
61%	60.2	23.3	11.9
68%	71.5	28.8	12.6
79%	96.4	39.4	14.4
90%	157.1	65.4	16.8

## Data Availability

The data presented in this study are available on request from the corresponding author.
